# Multi-pronged neuromodulation intervention engages the residual motor circuitry to facilitate walking in a rat model of spinal cord injury

**DOI:** 10.1038/s41467-021-22137-9

**Published:** 2021-03-26

**Authors:** Marco Bonizzato, Nicholas D. James, Galyna Pidpruzhnykova, Natalia Pavlova, Polina Shkorbatova, Laetitia Baud, Cristina Martinez-Gonzalez, Jordan W. Squair, Jack DiGiovanna, Quentin Barraud, Silvestro Micera, Gregoire Courtine

**Affiliations:** 1 Bertarelli Foundation Chair in Translational Neuroengineering, Center for Neuroprosthetics and Institute of Bioengineering, School of Bioengineering, Ecole Polytechnique Fédérale de Lausanne (EPFL), Lausanne, Switzerland; 2 Center for Neuroprosthetics and Brain Mind Institute, School of Life Sciences, Swiss Federal Institute of Technology (EPFL), Geneva, Switzerland; 3 Department of Clinical Neuroscience, Lausanne University Hospital (CHUV) and University of Lausanne (UNIL), Lausanne, Switzerland; 4 Defitech Center for Interventional Neurotherapies (.NeuroRestore), CHUV/UNIL/EPFL, Lausanne, Switzerland; 5 Motor Physiology Laboratory, Pavlov Institute of Physiology, St. Petersburg, Russia; 6 Neuromorphology Laboratory, Pavlov Institute of Physiology, St. Petersburg, Russia; 7 The BioRobotics Institute, Scuola Superiore Sant’Anna, Pisa, Italy; 8 Department of Neurosurgery, CHUV, Lausanne, Switzerland

**Keywords:** Brain-machine interface, Spinal cord injury

## Abstract

A spinal cord injury usually spares some components of the locomotor circuitry. Deep brain stimulation (DBS) of the midbrain locomotor region and epidural electrical stimulation of the lumbar spinal cord (EES) are being used to tap into this spared circuitry to enable locomotion in humans with spinal cord injury. While appealing, the potential synergy between DBS and EES remains unknown. Here, we report the synergistic facilitation of locomotion when DBS is combined with EES in a rat model of severe contusion spinal cord injury leading to leg paralysis. However, this synergy requires high amplitudes of DBS, which triggers forced locomotion associated with stress responses. To suppress these undesired responses, we link DBS to the intention to walk, decoded from cortical activity using a robust, rapidly calibrated unsupervised learning algorithm. This contingency amplifies the supraspinal descending command while empowering the rats into volitional walking. However, the resulting improvements may not outweigh the complex technological framework necessary to establish viable therapeutic conditions.

## Abstract (web summary)

Deep brain stimulation and epidural electrical stimulation of the spinal cord enable locomotion in humans with spinal cord injury (SCI) but the potential synergy between both approaches is unclear. The authors show that a complex technological approach is required to enable volitional walking in rats with SCI.

## Introduction

To initiate locomotion, the brain broadcasts motor commands that cascade through parallel neuronal pathways to converge onto executive centers residing in the lumbar spinal cord^1^. A spinal cord injury (SCI) disrupts the information flow within this well-organized communication system, which leads to locomotor deficits or even paralysis. However, the lumbar executive centers are usually distant from spinal cord damage. While anatomically intact, they are missing essential sources of modulation and excitation to produce locomotion^2,3^. Moreover, most SCIs spare some descending fibers with synaptic projections below the injury^4,5^. However, these anatomically intact projections remain functionally silent^2,6^. This understanding led to the development of neuromodulation treatments that tap into lumbar executive centers and residual descending projections to enable locomotion after SCI^7^.

For example, epidural electrical stimulation (EES) applied over the lumbar spinal cord^8–10^ instantly reestablished supraspinal control of leg movements in patients who had sustained an SCI leading to leg paralysis^8–11^. EES recruits proprioceptive afferents at their entrance in the spinal cord through the posterior roots^8,9^. The widespread projections of these afferents raise the excitability of spinal circuits to a level that enables residual descending projections to initiate leg movements. Studies in rodent models showed that the motor cortex relays the locomotor command to lumbar executive centers through glutamatergic neurons located in the gigantocellular nucleus^2^. After a clinically relevant model of contusion SCI, a subset of these reticulospinal fibers retains intact synaptic projections within the lumbar grey matter, which is sufficient to enable the motor cortex to control leg movements when EES is turned on.

Electrical currents have also been delivered within the midbrain using deep brain stimulation (DBS). The aim was to engage brainstem neurons with residual descending projections to lumbar execution centers^10^. Indeed, the midbrain contains an ancestral locomotor system that was first described functionally as a region that elicits locomotion when stimulated electrically^11,12^. A recent study segregated this region into two anatomically distinct nuclei that regulate specific locomotor behaviors^13^. The cuneiform nucleus (CnF) triggers high-speed locomotion to escape from danger. Instead, the pedunculopontine nucleus (PPN) controls slower locomotion underlying exploratory behaviors. DBS enhanced the vigor of locomotor movements after moderate SCI, and even enabled stepping-like movements after a SCI leading to paralysis^10^. Evidence indicated that this stimulation facilitates locomotion through the recruitment of glutamatergic reticulospinal neurons located in the gigantocellular nucleus^10^. These results suggest that, after SCI, the brain fails to engage the entire population of reticulospinal neurons with spared synaptic projections to lumbar executive centers.

These findings open the intriguing possibility to augment the facilitation of locomotion observed during EES with the additional stimulation of the midbrain locomotor region. Indeed, EES amplifies the ability of residual synaptic inputs from reticulospinal neurons to activate lumbar executive centers^2^. Consequently, the massive recruitment of reticulospinal neurons with DBS may increase the facilitation of locomotion enabled by EES. This putative synergy is appealing, since EES is highly effective in people with SCI^14–17^. Moreover, the delivery of DBS within the midbrain has mediated gait improvements in patients with Parkinson’s disease^18,19^, albeit the heterogeneity of responses and difficulties to position the electrodes in functionally relevant regions have hindered the widespread deployment of this method^20^. Indeed, a clinical trial is evaluating the therapeutic efficacy of DBS delivered in the midbrain of people with SCI^21^. Therefore, the simultaneous application of neuromodulation treatments targeting the midbrain and spinal cord may be a realistic strategy to improve locomotion after SCI in humans.

Here, we document the synergistic facilitation of locomotion when applying EES and DBS in a rat model of severe contusion SCI. However, we found that DBS mediated prominent signs of stress. Indeed, functional improvements were only observed with stimulation amplitudes that forced the rats into the undesired production of locomotion^12^. To remedy this issue, we linked DBS to the intention to walk, which we decoded from motor cortex activity using an unsupervised learning algorithm. This ecological^22^ brain-controlled delivery of DBS improved locomotion enabled by EES alone while alleviating stress responses.

## Results

### Implantation of DBS electrodes within the PPN to elicit locomotion

**Fig. 1:**
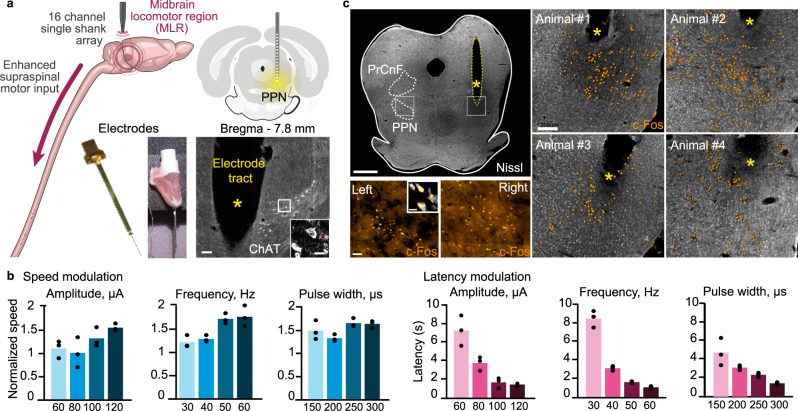
Targeted deep brain stimulation of the midbrain locomotor region (MLR). **a** Custom-made and commercial electrodes. Post-hoc anatomical evaluation of the electrode placement and localization of cholinergic (ChAT) neurons in the pedunculopontine nucleus (PPN), in the vicinity of the DBS electrode implantation site. PrCnF = Pre-Cuneiform Nuclei. Scales bar:, 100 μm (50 μm for inset). Repeated in *n* = 4 rats with similar results. **b** Locomotor speed modulation by MLR DBS as a response to different stimulation amplitude, frequency, and pulse width. Latency of locomotion initiation onset as a response to changing MLR DBS parameters (representative rat, *n* = 3 independent runs. Bars, data mean.). **c** Post-mortem reconstruction of midbrain tissues revealed that DBS induced a pronounced c-Fos expression in the vicinity of the electrode tips. Repeated in *n* = 4 rats with similar results in each (all shown). Scales bars: Left panel: 1mm, c-Fos 50 μm (inset 10 μm); Right panel 200 μm.

We aimed to deliver DBS in the midbrain to facilitate natural locomotor behaviors. Consequently, we targeted the PPN, which has been implicated in the production of basic locomotion^13^. For this purpose, we implanted custom-made or thin DBS electrodes with 16 contacts unilaterally in the left or right midbrain using stereotaxic coordinates corresponding to the center of the PPN of Lewis rats (AP = −7.8 mm ± 0.05, DV = −6.5 mm, ML = 2) (Fig. 1a). The delivery of DBS (40 Hz, 200 µs, 50–250 μA) elicited continuous locomotion with a latency of 5.78 ± 3.8 s (Fig. 1b). As repeatedly reported^12^. Increasing the frequency, amplitude, or pulse width of DBS progressively increased the speed of locomotion and reduced the latency between the onset of DBS and the initiation of locomotion (Fig. 1b and Supplementary video 1).

Post-mortem reconstruction of midbrain tissues revealed that the electrodes were inserted within PPN, which we localized based on clusters of cholinergic neurons^13^ (Fig. 1a). To verify the activation of PPN neurons during DBS, we visualized the expression of the immediate early gene fos in the midbrain following the delivery of DBS during 15 minutes (20 s on/10 s off), and terminated the rats 60 minutes later. These experiments were conducted under light anesthesia to prevent fos activation from neural activity not directly linked to the DBS per se, as would occur during walking or when rats move around in the cage. We found that unilateral DBS induced a pronounced, bilateral expression of the protein c-Fos in neurons located within the PPN (Fig. 1c), thus indicating that the unilateral stimulation targeted neurons located in the left and right PPNs to elicit locomotion.

### Functional impact of serotonin agonists, EES, DBS, and their combination

**Fig. 2:**
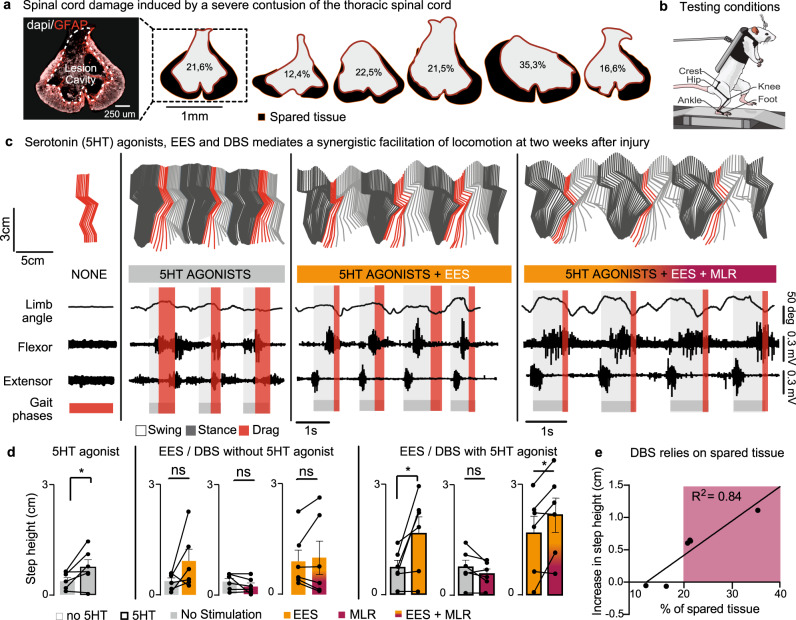
Impact of serotonin agonists, EES and DBS to enable stepping. **a** Representative photograph of the lesion epicenter, and graphical representation of sections with maximal damage for each rat from this group (*n* = 6 rats, all shown). Scale bar, 250 μm. The percentage of spared tissue is reported in each section. **b** Rats are tested at two weeks post-injury during stepping on a treadmill while supported in a robotic body weight support system. **c** Representative stick diagram decomposition of leg movements, oscillation of the whole limb (virtual limb linking the hip to the toe) and EMG activity of ankle muscles recorded at 2 weeks post-SCI with and without DBS. **d** Bar plots reporting mean values (*n* = 6 rats) of step height for each condition of stimulation. *, *P* < 0.05 using paired, one-tailed t-test (no 5HT, no stim vs 5HT, no stim, *p* = 0.043; 5HT, no stim vs 5HT + EES, *p* = 0.023; 5HT + EES vs 5HT, EES + MLR, *p* = 0.025); ns, not statistically significant. Bar plots, mean ± s.d. **e** Relationship between lesion size and increase in step height in the presence of both continuous EES + MLR and serotonergic agonists.

We modeled a severe contusion SCI using robotically controlled impacts (250 kdyn) onto midthoracic (T8/T9) spinal segments in several groups of rats (Fig. 2a). This lesion-induced variable white matter sparing that we characterized for each rat (Fig. 2a and Supplementary Table 1). In the same surgery, we implanted electrodes over the dorsal aspect of spinal segments L2 and S1 to deliver EES. One week after injury, all rats showed flaccid leg paralysis (Supplementary video 1).

We conducted a series of experiments to evaluate the specific and synergistic impact of serotonergic agonists, EES and DBS on the production of locomotion.

We first performed recordings during bipedal stepping on a treadmill during the early phase after the SCI (14 days post-injury, Fig. 2b), when the lumbar spinal cord below the injury is profoundly depressed^3^. Due to the complete paralysis, the effects of stepping-enabling interventions are best captured with a simple metric that distinguishes stepping versus not stepping. We therefore selected the change in step height as the primary outcome of this screening.

We reactivated the lumbar executive centers with a systemic administration of agonists to serotonergic receptors (5HT_1A_, 5HT_2A_, 5HT_7_)^3^. This serotonergic pharmacotherapy enabled weak, yet continuous stepping movements on the treadmill (Fig. 2c). In contrast, EES alone or DBS alone were ineffective or marginally effective to promote locomotion at this stage (Fig. 2d).

We then asked whether EES and DBS could increase the vigor of locomotor movements enabled by the serotonergic pharmacotherapy. Continuous EES (40 Hz, 300 µs, 100–300 μA)^23^ promoted a clear improvement of locomotion (Fig. 2c). For example, the addition of EES led to a significant increase in step height (*P* < 0.05, Fig. 2d). The impact of DBS depended on the severity of the injury. In rats with large spinal cord damage, the addition of DBS did not mediate changes in leg movements, both without and with EES and serotonergic agonists. When the SCI spared at least 20% of white matter tracts (*R*^2^ = 0.84, Fig. 2e), DBS did mediate improvement in the vigor of locomotion (Fig. 2c). However, this facilitation was contingent on the presence of both continuous EES and serotonergic agonists (Fig. 2d). Contrary to DBS, the facilitation of gait with EES was not correlated with the amount of spared tissue (*R*^2^ = 0.21, *P* = 0.4).

These results uncovered synergies between neuromodulation treatments targeting serotonergic receptor-bearing neurons, large-afferent fibers projecting to lumbar spinal cord, and reticulospinal fibers projecting to the lumbar spinal cord; although the facilitation of locomotion during the activation of reticulospinal neurons depended on the relative amount of spared white matter, as well as the presence of both serotonergic agonists and EES.

### Rehabilitative training augments the therapeutic efficacy of DBS

The relationship between the amount of spared white matter and functional impact of DBS was expected. Indeed, previous studies showed that the facilitation of locomotion with DBS relies on residual synaptic inputs from reticulospinal pathways to lumbar execution centers located below the injury^10^. We previously showed that robot-assisted rehabilitative training enabled by serotonin agonists and EES leads to a sprouting of descending pathways, in particular of reticulospinal neurons projecting to the lumbar spinal cord^2,23^. We thus reasoned that rehabilitative training could augment the functional impact of DBS on the facilitation of locomotion.

**Fig. 3:**
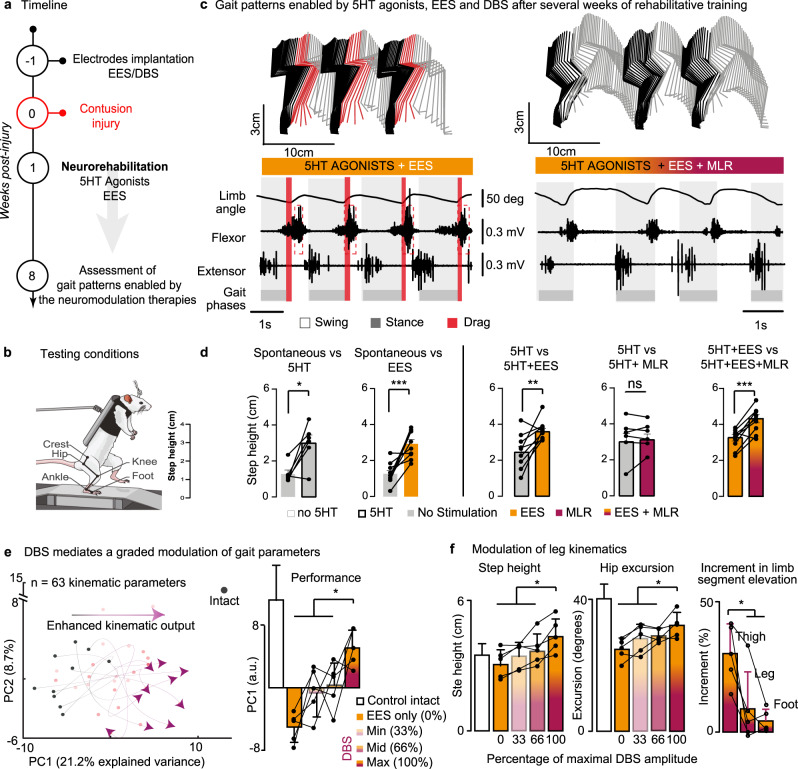
Rehabilitative training improves the impact of DBS on locomotor performance. **a** Timeline summarizing the experiments. **b** Rats are tested at 8 weeks post-injury during stepping on a treadmill while supported in a robotic body weight support system. **c** Representative stick diagram decomposition of leg movements, oscillation of the whole limb (virtual limb linking the hip to the toe) and EMG activity of ankle muscles recorded at 5 weeks post-SCI with 5HT agonists + EES versus 5HT agonists + EES + DBS. **d** Bar plots reporting mean values (*n* = 10 rats over 2 independent experiments) of step height for each condition of stimulation. *, *P* < 0.05, ** *P* < 0.01, ***, *P* < 0.001 using paired, one-tailed t-test (no 5HT, no stim vs 5HT, no stim, *p* = 0.009; no 5HT, no stim vs no 5HT, EES, *p* = 0.0004; 5HT, no stim vs 5HT + EES, *p* = 0.0046; 5HT + EES vs 5HT, EES + MLR, *p* = 0.0004). Error bars, s.d. **e** Principal components (PC) analysis (*n* = 5 rats). Gait patterns are displayed in the new reference frame created by PC1-2. The lines interpolate dots quantifying locomotor performance for the same rat in each of the four experimental conditions: EES and different levels of DBS intensities. The bar graphs report the scores on PC1 across conditions, which captured the enhancement of locomotor performance with DBS (gait patterns move in the direction of steps without injury). *, *P* < 0.05, ** *P* < 0.01, ***, *P* < 0.001 using paired, one-tailed t-test (0–100%, *p* = 0.0004; 33–100%, *p* = 0.004; 66–100%, *p* = 0.011). **f** Bar plots reporting mean values (*n* = 5 rats) of step height (0–100%, *p* = 0.0004; 33–100%, *p* = 0.004; 66–100%, *p* = 0.011) and hip excursion (0–100%, *p* = 0.0016; 33–100%, *p* = 0.0087; 66–100%, *p* = 0.039). The bar plot on the right reports the relative increase in the amplitude of the oscillations of each lower limb segments compared to EES only (Thigh-leg, *p* = 0.011; Thigh-foot, *p* = 0.0017). Paired, one-tailed t-tests. *, *P* < 0.05; **, *P* < 0.01; ***, *P* < 0.001. Bar diagrams, mean ± s.d.

Rats (Supplementary Table 1) were trained^2,23^ to step on a treadmill and overground towards a reward located at the end of a linear runway while supported bipedally in a multidirectional bodyweight support system^24^. Serotonin agonists and EES were administered to reactivate lumbar execution centers during training (Fig. 3a). After a few weeks of training, rats positioned bipedally on a treadmill (Fig. 3b) displayed weak spontaneous locomotor movements that extinguished rapidly. Serotonergic agonists alone and EES alone promoted continuous, coordinated stepping movements, albeit the more powerful locomotion was observed when both interventions were delivered simultaneously (Fig. 3c). The additional delivery of DBS mediated clear improvement of locomotor performance (Fig. 3c and Supplementary video 1). This facilitation required electrical currents with amplitudes comparable to those effective to trigger the initiation of locomotion before the injury (74±23 μA versus 84±42 μA, before versus after SCI). DBS alone failed to facilitate locomotion in these rats (Fig. 3d).

To quantify the relative degree of improvement, we applied a principal component analysis on a comprehensive set of 63 variables calculated from kinematic recordings (Supplementary Table 2). In all tested rats, we found that locomotor improvements increased with the intensity of DBS. At the optimal intensity (100%), the rats displayed large excursions of all lower limb joints with elevated step heights without paw dragging, which translated into vigorous stepping patterns (Fig. 3e, f).

These experiments showed that rehabilitative training enabled by serotonergic agonists and EES, which are known to mediate a pronounced sprouting of reticulospinal projections into the lumbar spinal cord below a contusion SCI^2^, augmented the beneficial impact of DBS on the production of locomotion.

### DBS facilitates voluntary locomotion, but triggers stress responses

**Fig. 4:**
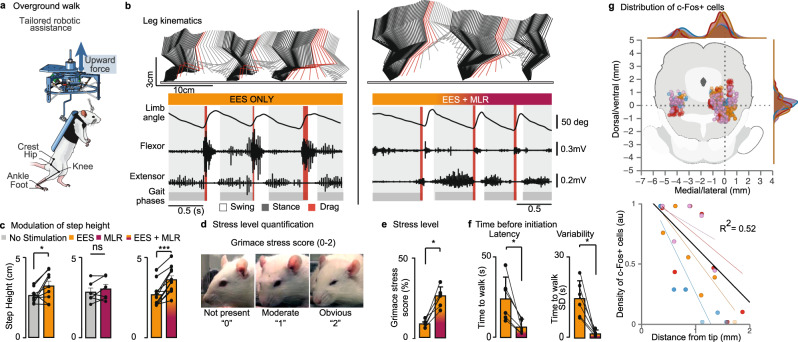
DBS improves locomotor performance during overground locomotion, despite stress responses. **a** Schematic representation of the testing conditions: bipedal locomotion along a runway with tailored robotic assistance. All the evaluations are performed with EES and 5HT agonists. **b** Same representations are in Fig. 2 during overground locomotion. **c** Bar plots reporting the mean values (±s.d) of step height under the different conditions (No stim vs EES, *n* = 9 rats, *p* = 0.025; No stim vs MLR, *n* = 6 rats; EES vs EES + MLR, *n* = 11 rats, *p* = 0.0001 using paired, one-tailed t-test). **d** Quantification of stress responses using the Rat Grimace Scale, wherein grimaces are scored from 0 to 2, as exemplified on three photos of the same rat, from Sotocinal S et al. Mol Pain 2011 (CC-BY license). **e** Bar plots reporting the mean values of stress responses with and without DBS (*p* = 0.00086 paired, one-tailed t-test). **f** Bar plots reporting the mean values of latency and variability (*p* = 0.0156 and *p* = 0.0156 paired, one-tailed Wilcoxon signed-rank test) in the latency between the beginning of the trial and the initiation of locomotion with and without DBS (*n* = 6 rats). *, *P* < 0.05; **, *P* < 0.01; ***, *P* < 0.001. ns, not statistically significant. Bar diagrams, mean ± s.d. **g** Top: Distribution of c-Fos+ neurons in the midbrain following DBS stimulation. Each color refers to a different rat (*n* = 4 rats). The plot shows the relationships between the number of c-Fos+ neurons and the distance from the tip of the electrode for each rat.

After a few weeks of training, all the rats with at least 10% of spared white matter tracts (*n* = 11 out of 13 trained rats) had regained the ability to initiate and sustain continuous locomotion overground in the presence of EES and serotonin agonists (Fig. 4a-b). Contrary to automated stepping on a treadmill, overground locomotion requires active participation from supraspinal centers^23^.

While rats were able to produce locomotion voluntarily, they typically dragged their paw at the beginning of the swing phase. This paw dragging led to an over-activation of the excessively stretched flexor muscles of the ankle. The delivery of EES and DBS led to significant improvements of locomotor performance, as observed on the treadmill (*p* < 0.05; Fig. 4c). However, we noticed that the delivery of DBS triggered stress responses and abnormal behaviors in all tested rats.

We quantified these responses using the grimace scale^25^(Fig. 4d). Rating of stress responses by two independent blinded observers revealed that DBS increased the stress level by 210 ± 103% (Fig. 4e). Adjusting the amplitude of DBS did not alleviate these responses, since the discomfort appeared around the threshold necessary to mediate the facilitation of locomotion. These observations were repeated twice, in two distinct groups of rats that were tested by different teams of investigators. We noticed that the delivery of DBS triggered a fast and systematic initiation of locomotion. This response contrasted with the relatively slow and variable time to initiation of self-initiated locomotion with EES alone (Fig. 4f). We concluded that DBS forced the rats to engage in the locomotor behaviors, which may explain the stress responses.

Alternatively, or complementarily, the discomfort might have arisen from the undesired stimulation of adjacent neural structures. However, we found that the expression of c-Fos following DBS was restricted to neurons located around the tip of the electrodes, which were all nested within the PPN (Fig. 4g). Indeed, the c-Fos expression pattern extinguished rapidly when moving away from the PPN (Fig. 4g). These results suggest that a putative spread of current that would activate untargeted neural structures was unlikely to explain stress responses.

These combined results show that the delivery of DBS augments locomotor performance when EES enables rats to produce volitional locomotion after a severe SCI. However, DBS forced the rats into the production of locomotion, which leads to an undesired behavior and thus substantial stress responses that would likely preclude the use of DBS in prosthetic applications.

### Locomotor-related cortical neurons are activated prior to midbrain neurons

To alleviate these stress responses, we sought to enable the rats to trigger DBS only when they desired to walk. Neurons located in the leg region of the rat motor cortex (MI) anticipate the initiation of locomotion^26^. We asked whether this increase in the activity of MI neurons precedes the activation of neurons located in the midbrain locomotor region. We reasoned that decoding of the intention to walk from MI recordings could trigger the onset of DBS.

**Fig. 5:**
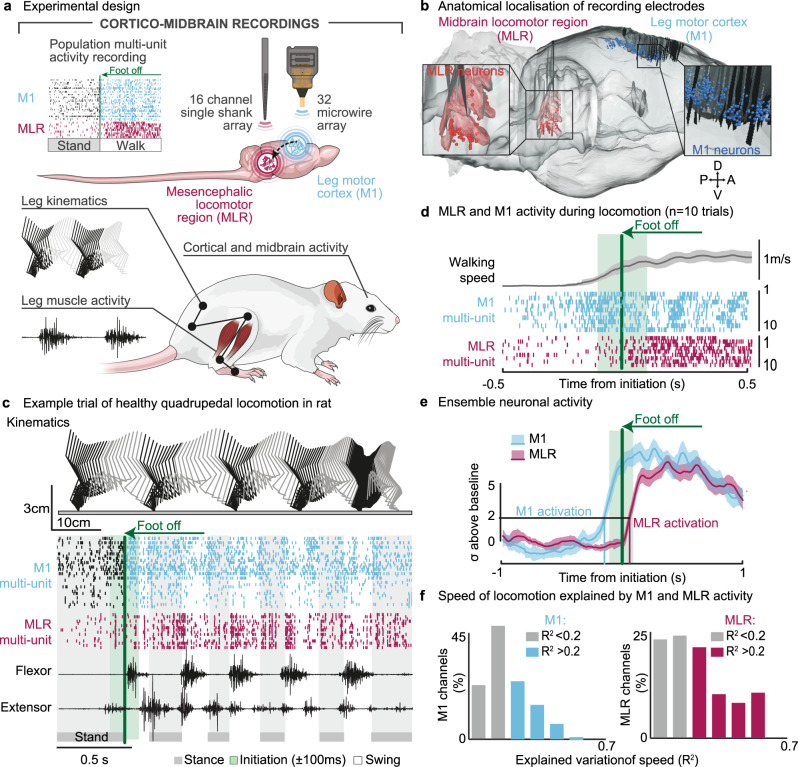
Encoding of locomotion in motor cortex and midbrain neurons in healthy rats. **a** Experimental setup depicting neuronal recordings from right MI (32 channels) and left MLR (16 channels) regions, together with whole body kinematics and muscle activity. **b** 3D visualization of MI and MLR electrode locations, combining in all experimental rats (*n* = 6 rats). A-P, anteroposterior; D-V, dorso-ventral. **c** Stick diagram decomposition of left hindlimb movement together with EMG activity of ankle muscles and multiunit activity recorded from MI and MLR regions. **d** Representative channel recorded from the electrodes located in MI and MLR with locomotor-related modulation of activity. The raster displays 10 successive trials. Shaded area, s.e.m. **e** Normalized averaged activity from all locomotor-related MI and MLR multi-units during gait initiation. Shaded area, s.e.m. **f** Distribution of MI and MLR channels encoding the speed of locomotion based on their relative correlation (*R*^2^) with average speed (*n* = 7 rats).

Healthy rats were implanted with electrodes in the leg region of the right MI and left midbrain to record the spiking activity of neurons, and with bipolar electrodes into selected leg muscles to monitor EMG activity (Fig. 5a, b). Analyses of neurons located in MI and midbrain revealed that both populations displayed an increase in firing rate prior to and throughout the production of self-initiated quadrupedal locomotion along a linear runway (Fig. 5c, d). To evaluate the relative timing at which each neuronal ensemble population anticipated the initiation of locomotion, we calculated a synthetic variable from all the firing rates of all the identified units that showed locomotor-related increase in activity. We considered that a given population was activated when the combined firing rates crossed a threshold of 2 standard deviations above the baseline.

With this criterion, MI activity preceded the initiation of locomotion by 147 ± 63 ms (*n* = 7 rats). We found that with our criterion, the activation of neurons located in the midbrain locomotor region occurred 104 ± 40 ms after gait initiation (*P* < 0.001) (Fig. 5e). The relative activation of these neurons was proportional to the speed of locomotion^27^ (*R*^2^ > 0.2 for 53% of the recorded channels, Fig. 5f).

These results show that neurons located in MI are activated prior to neurons in the midbrain locomotor region when healthy rats initiate locomotion voluntarily.

**Fig. 6:**
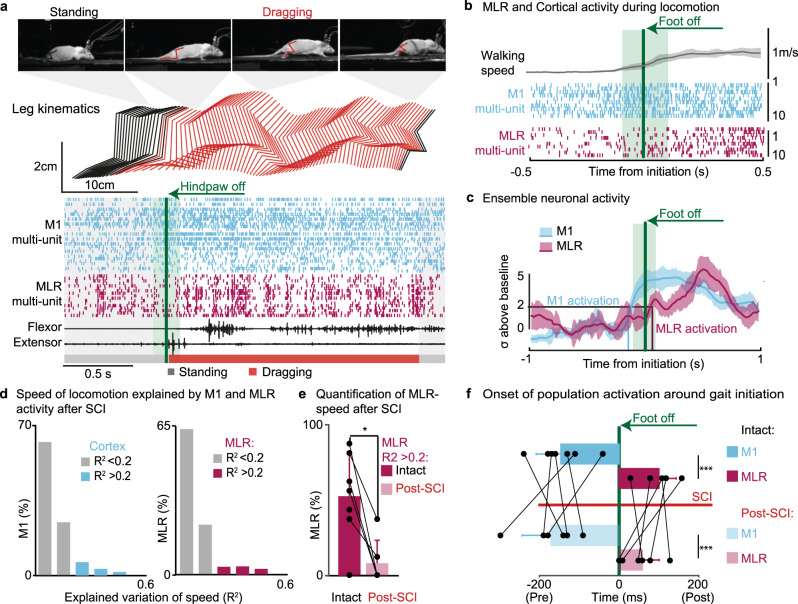
Encoding of locomotion in motor cortex and midbrain neurons in rats with SCI. **a** Sequence of photo showing quadrupedal locomotion after SCI, together with a stick diagram decomposition of left hindlimb movement, EMG activity of leg muscles and multiunit activity recorded from MI and MLR regions. (**b**) and (**c**) show the same representations as in Fig. 4 for recorded performed 2–3 weeks after the contusion SCI. Shaded areas, s.e.m. **d** Distribution of MI and MLR channels encoding the speed of locomotion based on their relative correlation (*R*^2^) with average speed (*n* = 6 rats). **e** Bar plot reporting the relative number of multi-units encoding the speed of locomotion in intact rats and after SCI (*n* = 6 rats, *p* = 0.031 paired, one-tailed Wilcoxon signed-rank test). **f** Bar plots reporting the mean values of the time at which the firing rates of all locomotor-related multi-units from MI and MLR crossed a threshold of 2 standard deviations above the baseline. The time is shown for all rats, before (*n* = 7 rats, *p* = 0.0001) and after SCI (*n* = 6, *p* = 0.00066). One-tailed Wilcoxon signed-rank test. *, *P* < 0.05; **, *P* < 0.01; ***, *P* < 0.001. Bar diagrams, mean ± s.d.

We then asked whether a severe contusion SCI disrupted the sequential activation of these two regions. After SCI, self-initiated quadrupedal locomotion along a runway was associated with a high level of variability in the firing rates of neuronal ensemble population located in the MI and midbrain (Fig. 6a–c). For example, the relative activation of neurons located in the midbrain failed to capture the well-known^27^ speed profile of the locomotor behavior (Fig. 6d, e and Supplementary Figure 1). Despite this variability, locomotor-related increase in the activity of MI neurons still anticipated the activation of neurons located in the midbrain when initiating locomotion. The temporal delay between the activation of both neuronal ensemble populations remained within the same range as before the SCI (182 ± 71 ms before gait initiation versus 55 ± 47 ms after gait initiation; Fig. 6f).

These results suggested that the intention to walk could be decoded from MI activity in order to trigger a more natural recruitment of the midbrain locomotor region with DBS, which may alleviate the stress responses observed when DBS is not synchronized with the intention to walk.

### Unsupervised learning algorithms decode the intention to walk

We next sought to develop an algorithm that can decode the intention to walk in real-time from MI activity without the need of learning procedures and long-lasting daily recalibration.

For this purpose, we implemented an unsupervised learning algorithm that aimed to distinguish between walking and idle states. After a few minutes of calibration, the decoder detected these states with 87.9 ± 8.4% and 90.6 ± 3.5% accuracy, respectively (Supplementary Fig. 2a). The intention to walk (activity prior to the first foot-off) involved a rapid transient in the activity of MI, which resulted in a sharp increase in the probability of transition to the walking state. Conversely, the termination of walking (last foot strike) correlated with a slow transient of detection probability (Supplementary Fig. 2b).

### Brain-controlled DBS augments performance while limiting stress responses

**Fig. 7:**
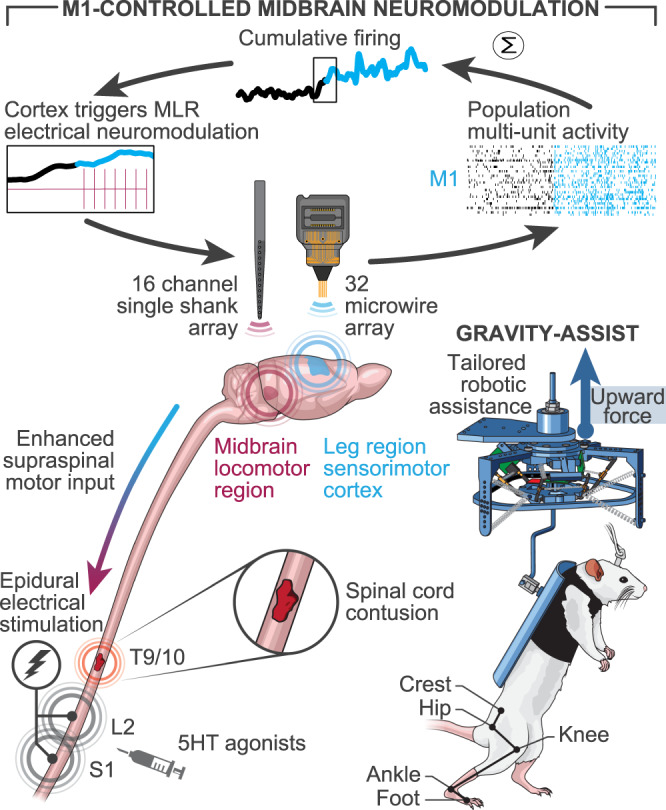
Neuroprosthetic system enabling volitional control of neuromodulation procedures. Recording of multiunit activity from MI is transformed into a synthetic variable that combines the firing of all locomotor-related multiunit, named cumulative firing. The cumulative firing is fed to an unsupervised algorithm that decodes walk and idle stages to turn MLR stimulation on and off. EES is continuously applied to the lumbosacral spinal cord in the presence of 5-HT agonists. Rats can initiate and sustain bipedal locomotion with a tailored gravity-assist, supported in a robotic body weight support.

We finally tested the possibility to link the intention to walk decoded from MI activity to the onset of DBS while the rats were spontaneously walking bipedally overground under continuous EES and agonists to serotonergic receptors (Fig. 7 and Supplementary video 1).

**Fig. 8:**
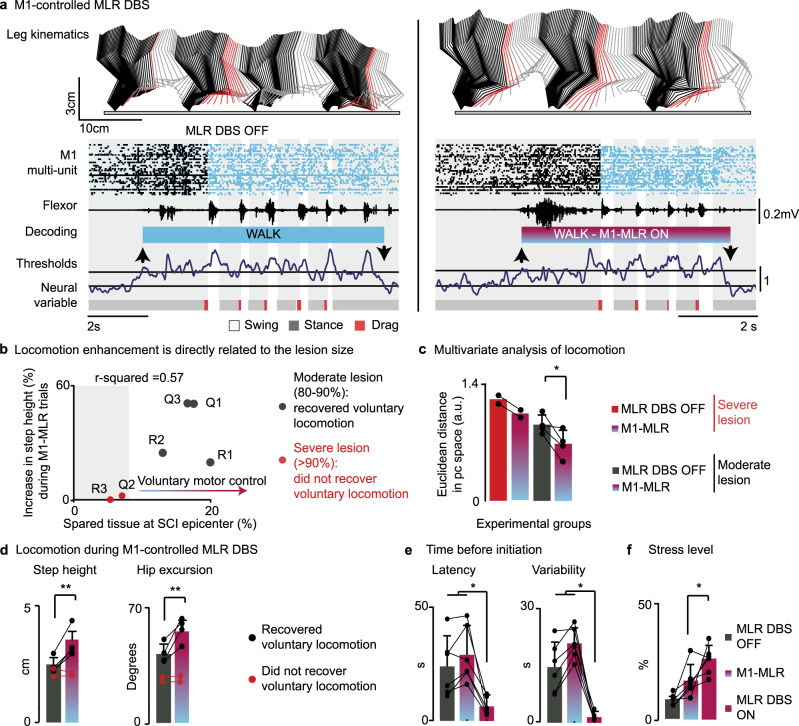
Brain-controlled MLR DBS improves locomotor performance while alleviating stress responses. **a** Same representation as in Fig. 5 during locomotion without DBS and with brain-controlled MLR DBS (M1-MLR). **b** Relationship between lesion size and increase in step height during brain-controlled MLR DBS. **c** Bar plots reporting the mean values of the Euclidian distance in the PC space between the different experimental groups (*n* = 4 rats, with additional *n* = 2 rats which did not recover voluntarily locomotion separately represented; *p* = 0.027, paired, one-tailed t-test). **d** Bar plots reporting mean values of step height and hip excursion (*p* = 0.0042 and *p* = 0.0088, paired, one-tailed t-test) without and with brain-controlled MLR stimulation (*n* = 4 rats, with additional *n* = 2 rats separately superimposed). **e** Bar plots reporting the mean values (*n* = 6 rats) of latency (MLR OFF vs ON, *p* = 0.016; M1-MLR vs MLR ON, *p* = 0.016; paired, one-tailed Wilcoxon signed-rank test) and variability (MLR OFF vs ON, *p* = 0.016; M1-MLR vs MLR ON, *p* = 0.016; paired, one-tailed Wilcoxon signed-rank test) in the latency between the beginning of the trial and the initiation of locomotion without MLR stimulation, with MLR DBS and with brain-controlled MLR DBS, and (**f**) associated mean values of stress responses under the same conditions (M1-MLR vs MLR ON, *p* = 0.043; paired, one-tailed t-test). *, *P* < 0.05; **, *P* < 0.01; ***, *P* < 0.001. Bar diagrams, mean ± s.d.

After 1 month of gait rehabilitation, four out of the six trained rats were able to initiate and sustain locomotion overground. Analyses of spinal tissues from the two rats that failed to regain voluntary locomotion revealed a more severe interruption of white matter tracts (Fig. 8b and Supplementary Table 1). In the four other trained rats, MI-controlled DBS improved locomotor performance compared to EES and serotonergic agonists alone (*p* = 0.03; Fig. 8a, c and Supplementary video 1). For example, this paradigm led to a 42% increase in step height associated with a 34% enhancement of hip excursions (Fig. 8d). The extent of these improvements was correlated with the amount of spared tissue (Fig. 8b; *R*^2^ = 0.57 and *R*^2^ = 0.5, respectively).

When MI activity triggered the onset of DBS, the latency and variability of the time before spontaneous gait initiation was similar to the condition without DBS (29.1 ± 12.2 s versus 23.9 ± 13.2 s) (Fig. 8e). This long latency contrasted with the rapid onset of locomotion when DBS was activated by the experimenter (5.5 ± 2.6 s), suggesting that MI-controlled DBS enabled a more natural, self-paced initiation of locomotion. Accordingly, rats displayed a significant decrease in stress level when MI triggered DBS compared to DBS alone (*p* < 0.05; Fig. 8f; −44.7 ± 39.7 %, decrease of 8.8 ± 10.1 points on the grimace scale).

## Discussion

We found that the concomitant delivery of electrical stimulation in the midbrain locomotor region and to the lumbar spinal cord promotes a synergistic facilitation of locomotion in otherwise paralyzed rats with severe contusion SCI. However, the stimulation delivered in the midbrain locomotor region was only effective when a sufficient amount of white matter tracts was spared, and that spinal cord neuromodulation treatments raised the excitability of lumbar executive centers. Moreover, the combination of interventions was comfortable for the rats only when the onset of electrical stimulation in the midbrain was synchronized with the intention to walk, which we detected from motor cortex activity using a rapidly calibrated, unsupervised learning algorithm. We discuss the putative mechanisms underlying the synergistic facilitation of locomotion with these combined neuromodulation treatments and consider the limited impact of these interventions for viable clinical applications.

### Engaging the residual motor circuitry after SCI

Imaging^28^, electrophysiological^29,30^, and anatomical^5^ evaluations showed that the majority of SCI spare bridges of intact neural tissues. These bridges embed descending nerve fibers that maintain synaptic projections within the spinal cord below the SCI. While anatomically intact, these spared fibers remain functionally silent^31^. EES can amplify the impact of synaptic inputs from these spared fibers onto lumbar executive centers^2^. When EES is turned on, individuals with SCI can immediately regain graded control over the activity of otherwise paralyzed muscles^14–17^. After several months of gait rehabilitation, EES enabled non-ambulatory individuals with chronic SCI to walk overground^15,16^ and adjust the movements of their legs to accommodate varying speeds and obstacles^14^. Here, we modeled this recovery in rats with severe midthoracic contusions that spared approximately 15% of white matter tracts. We show that gait rehabilitation facilitated by EES enabled otherwise paralyzed rats to regain the ability to initiate and sustain overground locomotion.

Previous studies conducted with this preclinical model revealed that the motor cortex orchestrates this recovery^2,23^. Gait rehabilitation enabled by EES promotes activity-dependent growth of new motor cortex projections into the brainstem regions containing reticulospinal neurons with spared connections to lumbar executive centers. In turn, these spared fibers sprout into lumbar grey matter regions that are relevant for motor control, which augments the strength of the connections between the brain and spinal cord^2,23^. After training, rats are able to transform contextual information into task-specific motor commands that are transferred to the spinal cord through de novo cortico-reticulo-spinal relays^2^. Importantly, silencing the synaptic release of glutamatergic reticulospinal neurons with spared synaptic projections to the lumbar spinal cord abolishes this recovery.

It is well established that the midbrain locomotor region engages lumbar executive centers through the recruitment of reticulospinal neurons^10,12,13,32^. We thus surmise that the recruitment of reticulospinal neurons with spared synaptic projections below the SCI accounted for the facilitation of locomotion observed in the present study when delivering electrical stimulation in the midbrain. Inactivation experiments have confirmed the contribution of this mechanism after less severe SCI^10^. This interpretation is also consistent with the temporal course of our experimental results. Indeed, stimulation of the midbrain locomotor region became effective only when the rats had regained the ability to initiate and sustain locomotion. The recovery of volitional locomotion coincides with the growth of new collaterals from reticulospinal fibers into the lumbar grey matter^2^. Thus, we propose that the stimulation of the midbrain locomotor region led to the synchronized activation of the near entire population of reticulospinal neurons with spared projections to lumbar executive centers. The robust activation of the immediate early gene fos in large clusters of neurons of the midbrain locomotor regions during DBS is also consistent with this interpretation.

Why does the brain fail to the engage the entire population of reticulospinal neurons voluntarily after SCI? Various studies reported that the interruption of descending and ascending pathways within the spinal cord propagates dysfunction throughout the central nervous system, even in distant regions located above the injury^10,33–35^, including the motor cortex^36–39^. For example, we found that the SCI disrupted the encoding of locomotor speed in motor cortex and midbrain neurons. These secondary changes in circuit dynamics alter the ability of residual fibers from projection neurons to contribute to the production of movement after SCI^2,10,40^. Such alterations may reduce the capability of supraspinal centers to engage reticulospinal neurons, thus explaining the additional facilitation of locomotion observed when delivering stimulation in the midbrain. However, it is worth noting that this facilitation required high-levels of electrical currents. As proposed above, such suprathreshold stimulation may lead to the massive recruitment of the entire population of reticulospinal neurons. However, the synchronized recruitment of a neuronal ensemble population is uncommon in the natural physiological operations of the central nervous system. For example, even when the integrity of descending control systems is not comprised, humans are not able to recruit an entire pool of motor neurons simultaneously^41^. It is always possible to recruit additional motor units when applying electrical stimulation to efferent nerve fibers. While the relative capacity to activate motor neuron pools can improve with strength training, this capacity never reaches 100%^42^. Similarly, we suspect that the ability of the motor cortex to engage reticulospinal neurons with spared synaptic projections below the SCI may improve during training, but never reaches the maximal activation that can be obtained with suprathreshold stimulation.

The non-physiological, synchronized activation of reticulospinal neuronal ensemble population had a detrimental impact on rats’ behavior. Indeed, the stimulation led to prominent stress responses in all tested rats. The undesired recruitment of nearby regions may be in part responsible for these detrimental side-effects. However, we found that the neurons directly activated by DBS were restricted to the region surrounding the electrodes, which were nested within the PPN. Moreover, stress responses vanished when DBS was coupled with the intention to walk. We concluded that the forceful engagement into non-volitional locomotion was the main reason for the observed stress responses.

To alleviate these undesired side effects of the stimulation, we linked the onset of the treatment with the intention to walk. Concomitant recordings of neurons in the motor cortex and midbrain locomotor region revealed that gait-related activation of the hindlimb motor cortex occurred before the recruitment of midbrain neurons. We thus decoded the intention to walk or rest from motor cortex activity. We conceived a robust decoder based on unsupervised learning algorithms that only required a few minutes of calibration to couple the intention to walk or rest to the onset or end of midbrain stimulation. This decoder can be readily implemented to drive other types of gait neuroprostheses^43–45^. This more ecological^22^ delivery of neuromodulation treatments alleviated the stress responses. Moreover, the functional coupling between locomotor-related motor cortex activity and midbrain stimulation reduced the amount of electrical current necessary to observe maximal improvements in locomotor function, suggesting a synergistic activation of reticulospinal neurons.

### Therapeutic requirements and clinical perspectives

Multiple independent research groups have reported the ability of EES to restore voluntary control of leg movements in humans with SCI^14–17^. Various studies also documented the facilitation of locomotion when DBS is delivered in the PPN of people with Parkinson’s disease^18,19^, albeit independent studies have reported heterogeneity in patient responses ^46^. The variable position of the electrode contacts may explain this heterogeneity. Indeed, the functionally relevant regions of the brainstem to improve gait remain difficult to identify in humans. The most recent studies suggest that targeting the posterior PPN and cuneiform nucleus at the level of the pontomesencephalic junction would be the most effective localization to alleviate freezing of gait in patients with Parkinson’s disease^19,20^. This region coincides with the location that we targeted in rats, although the relatively small size of this area renders the comparison with humans difficult.

Regardless of the extent or consistency of the therapeutic benefits in people with Parkinson’s disease, these earlier clinical studies established an expedited path for preliminary evaluation of the efficacy of DBS delivered in the PPN of people with incomplete SCI^21^. Therefore, the simultaneous application of neuromodulation treatments targeting the midbrain and spinal cord may rapidly be tested in humans with SCI.

However, the relative improvement of locomotor performance observed in our model of severe contusion SCI was modest compared to the complexity of the engineering framework necessary to observe viable therapeutic gains. Moreover, side-effects of DBS delivered to the midbrain have been well documented in people with Parkinson’s disease^18,47,48^. Due to the pronounced destruction of reticulospinal tracts following spinal cord damage, the facilitation of locomotion with DBS would likely involve larger stimulation intensities in people with severe SCI compared to patients with Parkinson’s disease. Indeed, in our model of severe contusion SCI, detectable improvements in stepping required stimulation currents reaching the levels necessary to trigger non-volitional locomotion in healthy rats^10^. The midbrain is a heterogeneous region that modulates various physiological functions and embeds a broad diversity of intermingled neuronal pathways that may be recruited with large intensities of stimulation. While multipolar configuration with directional leads may mitigate these non-desired effects^46^, we suspect that substantial side-effects may be expected in order to reach intensities that mediate locomotor improvements in humans with severe SCI.

Nevertheless, it is possible that DBS would be effective at lower stimulation amplitudes in people with less severe SCI. We found that the relative impact of DBS strongly correlated with the relative amount of spared tissue. It is thus possible that with mild SCI severities, DBS would be effective at amplitudes that match the more acceptable levels of stimulation intensities delivered in the PPN of people with Parkinson’s disease to improve gait. Moreover, imaging methods are becoming available to identify the region of the rostral brainstem that is most likely to mediate functional benefits when delivering DBS^20^. Finally, the introduction of DBS during gait rehabilitation may augment the reorganization of descending pathways, in particular reticulospinal pathways that may be more robustly recruited during training with this intervention. This hypothesis will need to be evaluated in preclinical models of moderate SCI prior to clinical evaluations.

We conclude that after severe SCI, the combination of DBS delivered in the midbrain locomotor region and EES applied to the lumbosacral spinal cord may not mediate locomotor improvements that outweigh the complex engineering framework necessary to promote viable therapeutic effects. Therefore, we doubt that this pathway is viable clinically. However, we confirm the ability of midbrain DBS to augment the descending activation of lumbar executive centers, suggesting that this intervention might facilitate recovery after less severe SCI in humans, notably in combination with gait rehabilitation. Finally, the robust synergy between neuromodulation treatments delivered above and below a SCI requires further investigations, since alternative targets in the brain or brainstem may be better tolerated while mediating superior efficacy.

## Methods

### Study design

An initial pilot experiment (rats A1–A3; Supplementary Table 1) examined the effect of supplementing EES of lumbosacral segments with deep brain stimulation (DBS) of the midbrain locomotor region. Having observed a clear augmentation of stepping ability in these animals, as well as a clear stress response during MLR DBS, we conducted experiments in a larger cohort of rats (R1-4, Q1-3; Supplementary Table 1) to compare the effects of open-loop (“forced”) DBS and closed-loop (“M1-controlled”) DBS in combination with serotonin agonists and EES. All these experiments were conducted in the presence of serotonergic agonists. Therefore, we only compared the conditions of serotonin agonists + EES versus serotonin agonist + EES + DBS. To further break the effects of single interventions and their combination, we performed experiments with an additional cohort of rats that followed a rehabilitation training regimen enabled by 5HT agonists and EES (N1-6; Supplementary Table 1). Spontaneous stepping was always recorded first, to avoid fatigue. Due to the lasting effects of 5HT agonists, the conditions EES alone, MLR DBS alone, EES + DBS were recorded before the administration of 5HT agonists. Since DBS led to stress response, this condition was tested last in the sequence of experiments. All the recordings could be collected at 2 weeks post-injury. However, two rats lost EES electrodes in the chronic stage of SCI. Therefore, we utilized a final cohort of five rats (O1-5; Supplementary Table 1) to evaluate the condition spontaneous vs EES alone at this time point.

### Experimental setup

Experiments were conducted on adult female Lewis rats (200–220 g body weight). The rats were housed individually in transparent cages with access to food and water ad libitum. The room was kept on a 12 h light/dark cycle at 22 degrees Celsius ambient temperature. Prior to surgery, all the rats were handled and trained to freely walk along the runway. Animal care, including manual bladder voiding, was performed twice per day throughout the whole post-injury period. All experimental procedures were approved by the Veterinary Office of the Cantons of Vaud and Geneva, Switzerland.

### Surgical procedures and post-surgical care

The rats underwent two surgeries performed under aseptic conditions and general anesthesia: they were first implanted with electrodes into the brain, midbrain, spinal cord, and muscles. One week later, the midthoracic spinal cord was contused. Here, we describe each implant: following the production of two craniotomies, and the removal of the dura mater, two electrode arrays were implanted. A 32-channel microelectrode array (Tucker-Davis-Technologies, USA) was inserted into layer V of the hindlimb area of the right motor cortex (M1)^26^. A 16-channel single-shank multielectrode array (CM16LP, NeuroNexus, USA) was inserted in the left midbrain using the coordinates that correspond to the PPN in Lewis rats: AP = −7.9 mm ± 0.05, DV = −6.5 mm, ML = 2. Note that the anteroposterior position was adjusted to avoid blood vessels. Ground and reference wires from both arrays were attached to screws fixated to the skull. Bipolar intramuscular electrodes were inserted in the medial gastrocnemius (MG, ankle extensor) and tibialis anterior (TA, ankle flexor) muscles to record electromyographic (EMG) activity. Two stimulating wire electrodes were sutured to the dura over lumbar (L2) and sacral (S1) spinal segments. A common ground wire (~1 cm of Teflon removed at the distal end) was inserted subcutaneously over the right shoulder. Brain arrays, spinal and EMG wires connectors were all fixed using dental acrylic and microscrews secured to the skull for fixation. The contusion was delivered using a force-controlled impactor (IH-0400 Impactor, Precision Systems and Instrumentation LLC, USA) set at 250 kdyn (1 dyn = 10 μN) applied at the midthoracic level (approximately T8). The spinal segment was exposed through a skin incision, separation of the dorsal muscles and a laminectomy. The dura mater was not removed. Analgesia (buprenorphine Temgesic®, ESSEX Chemie AG, Switzerland, 0.01–0.05 mg per kg, s.c.) and antibiotics (Baytril® 2,5%, Bayer Health Care AG, Germany, 5–10 mg per kg, s.c.) were provided for 3 and 5 days post-surgery, respectively.

### Locomotor training

Rats were trained 5 days per week for 30 min per day starting from day 7 post-injury. Five minutes prior to each training session, the rats received an intraperitoneal injection of quipazine (5-HT2A/C, 0.2–0.3 mg/kg) and 8‐OH-DPAT (5-HT1A/7, 0.05–0.2 mg/kg) adjusted daily based on locomotor output. Locomotor training was performed by first positioning the rat bipedally on a treadmill moving at 11 cm/s with partial vertical support (Robomedica, USA), and later transferring the rat for training overground on a linear runway with a postural robotic interface^24^. The duration of the training started from 25 min on the treadmill and 5 min overground and was gradually adjusted to 5 min on treadmill and 25 min overground, depending on the animal’s performance. During all training sessions, monopolar stimulation pulses were delivered tonically at L2 and S1 electrodes (40 Hz, 50–350 µA, 0.2 ms).

### Kinematic and EMG recordings and analysis

Locomotor performance was evaluated during walking on the treadmill (11 cm/s) and along a straight runway. Electrical stimulation and optimal body weight support were provided as needed and maintained constant during all recorded conditions. The following conditions were recorded on the treadmill (with and without serotonergic agonists) and the runway (only with serotonergic agonists): no stimulation, EES stimulation, MLR DBS stimulation, and EES + MLR DBS stimulation. Kinematic (12 infrared and 2 digital video cameras, 200 Hz) and EMG recordings (2 kHz, 10–1000 Hz bandpass filtered) were performed using an integrated motion capture system (Vicon, UK)^49^. Reflective markers were attached at the iliac crest, greater trochanter, lateral condyle, lateral malleolus, distal end of the fifth metatarsal, and toe. Nexus (Vicon) was used to track the 3-D position of the markers. To quantify locomotor performance^50^, we isolated single gait cycles, extracted relevant kinematics parameters and applied a principal component (PC) analysis on 63 computed variables (See Supplementary Table 1).

### Evaluation and characterization of midbrain stimulation

Following recovery from electrode implantation, we tested behavioral responses to stimulation delivered in the midbrain locomotor region (40 Hz train of 200 μs long biphasic pulses, amplitude of 50–250 μA). Only rats that exhibited locomotion shortly after the onset of the stimulation were included in the study. We report the following implant success rates: Pilot animals, 3/3 functional with commercial 16-channel electrodes; This study, 13/13 functional (16-channel). One month after SCI, rats were positioned bipedally on a treadmill. Locomotion was recorded under pharmacological and EES, with DBS initially switched off. The intensity of DBS stimulation was defined within the range of amplitudes that were not unpleasant for the rat (no orbit tightening, no squeaking). The relative activation of the midbrain locomotor region was defined as 100% (max), 66% (intermediate), 33% (low) of the maximum amplitude to facilitate locomotion.

### Recordings of midbrain neurons

Extracellular voltage signals were pre-amplified, digitalized, sampled at 24 kHz and stored using a BioAmp processor (Tucker-Davis Technologies, USA). The channel average was subtracted offline from each trace to remove common mode noise. Multiunit activity (MUA) consisted of all field potential stochastic events that crossed the threshold value of three standard deviations of the potential signal. For all offline analyses, spike counts were binned using windows of 10 ms. To compute the encoding of locomotor speed, both traces were low pass filtered with a 200 ms moving average.

### Cortex-midbrain interface

Intracortical voltage signals were pre-amplified, digitalized and sampled at 24 kHz, then bandpass filtered online (0.7–3 kHz), all by means of a real-time BioAmp processor (Tucker-Davis Technologies, USA). Cortical multiunit activity (MUA) consisted of all field potential stochastic events that crossed a threshold value. Spike count was collected in bins of 10 ms and crossed a Finite Impulse Response (FIR) filter with Gaussian sample decay of 80% in 40 ms. A single trial of overground walking between the two extremes of the runway (quadrupedal or bipedal, robot-assisted) featured an approximately even balance of idle standing time and walking time (usually 5 to 10 s of data for quadrupedal walking and 10 to 40 s for bipedal). A Self-Organizing Map (SOM) with four output nodes (Matlab function selforgmap([4 1])) was used to segregate the cortical activity recorded during the calibration trial in four ordered clusters, based on the statistical properties of the cortical signal. A cluster value of one was assigned to the state featuring the highest mean spike count across the cortical population. The second was assigned 2/3, the third 1/3, while the last was assigned the value of 0.

A time vector **Y** was created (Y: 1x*t*), where each sample holds the value of the observed cluster. The **Y** vector was then smoothed with a moving window of 500 ms length. A linear combination *y* = **wn** of the MUA of all 32 channels was used as a normalized control variable in online testing (*y*: 1x1 current control variable value, **w**: 1 × 32 weights, **n**: 32 × 1 current MUA sample). The weight vector **w** was computed from data acquired during the calibration trial as the least square solution of **wN** = **Y**. Thus, **w** = **Y**(**N**)†, where (†) represents the Moore-Penrose pseudoinverse (**N**: 32 × *t* collection of MUA samples).

During online testing, whenever *y* = **wn** crossed a fixed detection threshold in the range 0.7–0.8, the real-time processor instantly delivered DBS through the electrodes located in the midbrain. Conversely, when *y* crossed a threshold positioned at a fixed value in the range 0.2–0.3, stimulation was turned off. The stimulation parameters were set at the maximal non-painful level identified during experiments on the treadmill. The acquisition and processing of data presented to the unsupervised learning algorithm typically required 5 min at the beginning of an experimental session. The hard-real-time program was run with cycles of 12 kHz.

### Raster plots

Spike occurrence time diagrams were obtained by offline analysis. Intracortical voltage data were bandpass filtered (700–3000 Hz) and z-scored. Each event crossing the threshold at −3 standard deviations was added to the neuronal spike count (multiunit activity).

### Immunohistochemistry and neuromorphological evaluation

Rats were deeply anesthetized by an i.p. injection of 0.5 ml Pentobarbital-Na (50 mg/mL) and transcardially perfused with approximately 80 ml Ringer’s solution containing 100 kIU/L heparin (Liquemin, Roche, Switzerland) and 0.25% NaNO2 followed by 300 ml of cold 4% phosphate buffered paraformaldehyde, pH 7.4 containing 5% sucrose. The brain and spinal cord were removed and post-fixed in the same fixative overnight and later transferred to 30% sucrose in phosphate buffer (PB) for cryoprotection. After 3 days, the tissue was embedded in Tissue Tek O.C.T (Sakura Finetek Europe B.V., The Netherlands), frozen at −40 °C, and cut to a thickness of 40 μm. For immunohistochemistry experiments, sections used for GFAP and Nissl staining were directly mounted, washed 3 times in 0.1M PBS and blocked in 10% (GFAP) normal goat serum containing 1% Triton. Sections were then incubated in primary antibody diluted in the blocking solution overnight at 4 °C (GFAP). The primary antibody used was rabbit anti-GFAP (1:1000, Dako, USA). Sections were again washed 3 times in 0.1M PBS and incubated with the appropriate secondary antibody (Alexa fluor® 488) in blocking solution. NeuroTraceTM (Life Technologies, USA) was used as a Nissl counterstain at a dilution of 1:50 in 0.1M PBS. Slides were finally washed, air-dried and coverslipped with Mowiol. To visualize the location of electrodes with respect to the PPN, we immunolabelled cholinergic neurons with antibodies against choline acetyltransferase (ChAT). First, the midbrains were sliced into series of 40 μm-thick slices. The tissue samples were then blocked on a shaker for 60 min in PBS 10% NDS and 0.3% Triton X100. Subsequently, the goat anti-ChAT primary antibody (1:100 in PBS 0.1M with 5% NDS and 0.3% Triton X100) was added overnight at 4 °C on the shaker. Finally, the donkey anti-goat secondary antibody (Alexa fluor^®^ 647, Life Technology A2 1432) was added at a dilution of 1:300 in 0.1M PBS with 3% NDS and 0.3% Triton X100 for 90 min at room temperature on the shaker. Lastly, slides were washed, air-dried and coverslipped with Mowiol.

### Evaluation of spinal cord damage

The extent and location of spinal cord damage were evaluated in each rat. The lesion cavity was cut in serial coronal sections (40 µm) that were stained using GFAP and Nissl staining. Spared tissue was measured using three fluorescent image stacks per rat, from the lesion epicenter going to the first rostral and caudal intact sections, acquired with Olympus Slide Scanner VS120-L100 microscope at 10x magnification and analyzed offline using custom-written Matlab scripts. Slide scanner output images were divided into square regions of interest (ROI). Files were color-filtered and binarized by means of intensity thresholds, set empirically and maintained across sections. Finally, the amount of tissue spared by the SCI was computed as the ratio of positive signal (amount of pixels) at the epicenter and the average positive pixel count at the intact sections.

### Evaluation of neuronal activation in the midbrain

Prior to perfusion, *n* = 4 rats from the study were lightly anaesthetized (0.75% gaseous isoflurane in medical O_2_) and then received MLR stimulation via their implanted electrode at 1.5x the intensity used for their final locomotor recordings (to account for increased threshold resulting from mild anesthesia). Frequency parameters remained the same as used throughout the study and stimulation was delivered in bursts of 20 s followed by a break of 10 s for a total of 15 minutes. Animals were perfused 60 minutes after the stimulation ended. The tissue was harvested and processed as described above. 40μm-thick cryosections of the midbrain electrode implantation site were blocked on a shaker for 60 min in PBS 10% BSA and 0.3% Triton X100. Subsequently, rabbit anti-c-Fos primary antibody (Synaptic Systems; 1:100 in PBS 0.1M with 5% BSA and 0.3% Triton X100) was added and left to incubate at room temperature for 48 hours on the shaker. Finally, goat anti-rabbit secondary antibody (Alexa fluor® 647, Life Technology) was added at a dilution of 1:500 in 0.1M PBS with 3% BSA and 0.3% Triton X100 for 90 minutes at room temperature on the shaker. Lastly, slides were washed, air-dried and coverslipped with Mowiol.

Image acquisition was performed using an Olympus slide scanner VS120-L100 microscope and images were processed offline using Imaris software (Bitplane, USA). C-Fos+ nuclei distribution was measured using the built-in spot detection algorithm in one representative section per animal at the location of the electrode tip. The threshold for spot detection was verified for each animal by 2 independent investigators to ensure a clear distinction of the c-Fos+ signal. Following segmentation, the density of c-Fos+ neurons from the electrode site was summed in 250 µm bins. Due to tissue damage, the bin closest to the electrode site was not included. The distance from the tip was calculated as distance *d* = sqrt(*X*^2^ + *Y*^2^), using the *XY* coordinates of the tip as reference for each animal. Units were then scaled between 0 and 1 to normalize between rats. Mixed model linear regression was used to examine the relationship between distance from the electrode tip and the density of c-Fos+ neurons according to the formula: density ~ distance + (1|rat_id), with a fixed effect for distance and a random effect for rat. Mixed model analyses were carried out using the lme4 package in *R*, and the corrected *R*^2^ calculated using the MuMIn package.

### Quantification of stress responses

Rats walked from the starting point to a horizontal bar located 50 cm in front of them. For each trial, a picture of the rat was taken at the exact moment it touched the horizontal bar. The level of stress was then evaluated using an adapted version of the Rat Grimace Scale (RGS)^25^. Specifically, we did not quantify the “whisker change” parameter since this component was difficult to identify from the lateral angle, where the picture could be taken. The pictures were then shuffled, blinded and presented to two independent evaluators who were not involved in the experiments. Each of the parameters: orbital tightening, nose/cheek flattening, whisker deflection, and posture were scored on the scale from 0 to 2, with 0 being normal and 2 indicating the most abnormal state, respectively. Observers’ scores were later analyzed and reported in percentage values and averaged across all rats (*n* = 6).

### Statistics

The number of rats per group was determined by power analysis on the basis of a pilot experiment on rats A1–A3 (Supplementary Table 1). We found that MLR stimulation on runway increased the leg trajectory and hip excursion by +50% of the range displayed spontaneously by the animals. Under this condition, and assuming 20 gait cycles per animal, a power analysis (using the non-parametric Wilcoxon signed-rank test) determined that we would have 75% chance to report a significant effect (α = 0.05) with *n* = 5 rats and 92% chance to report this effect with *n* = 6 rats. We repeated the main observations of the study two or three times, with independent groups of rats and different teams of investigators. For all the groups, we maintained the pre-determined sample of *n* = 6 rats. Conditions were not randomized. Due to the stressful responses induced by DBS, we always placed this condition at the end of the experimental recording sequence. This sequence mitigated the impact of DBS on the rats’ motivation to perform the task spontaneously.

Average cortical ensemble firing rates are reported as mean values ± SEM to display the distribution of the mean of the neural variable. All other data are displayed as mean values ± SD. Paired statistical evaluations were performed using Student’s *t* test or the non-parametric Wilcoxon signed-rank test when at least one of the populations could not be assumed to be normally distributed (after applying the one-sample Kolmogorov–Smirnov test). Tests are one-sided, as our hypotheses were always strictly defined towards the direction of motor improvement.

### Reporting summary

Further information on research design is available in the Nature Research Reporting Summary linked to this article.

## Supplementary information

Supplementary Information

Description of Additional Supplementary Files

Supplementary Movie 1

Reporting Summary

## Data Availability

Complete source data are provided with this paper. Data that support the findings will be made available upon reasonable request to the corresponding author.

## Source data

Source Data

## Code availability

Custom-made scripts used in this Article are available in a GitHub repository at https://github.com/multipronged-neuromodulation/M1-MLR
