# Supplementary material for: Multi-pronged neuromodulation intervention engages the residual motor circuitry to facilitate walking in a rat model of spinal cord injury
Source: Nat Commun. 2021 Mar 26;12:1925. doi: 10.1038/s41467-021-22137-9 (PMC7997909; doi:10.1038/s41467-021-22137-9)
Supplement: Supplementary file 1 — Supplementary Information [file 41467_2021_22137_MOESM1_ESM.pdf]

SUPPLEMENTARY MATERIAL

Animal ID	Spared tissue at epicenter (%)	Treadmill Acute MLR (Fig. 2)	Treadmill Chronic MLR (Fig. 3)	Runway Forced MLR (Fig. 4)	MLR Intact recording (Fig. 5)	MLR Intact recording (Fig. 6)	M1-controlled MLR DBS (Fig. 8)
A1	12.7		✓	✓			
A2	31.8		✓	✓			
A3	10.2		✓	✓			
R1	19.8		✓	✓	✓	✓	✓
R2	12.9		✓	✓	✓	✓	✓
R3	5.3		‡		✓	✓	✓&
R4	-		§		✓	§	
Q1	17.4		✓	✓	✓	✓	✓
Q2	7.0		‡		✓	✓	✓&
Q3	16.5		✓	✓	✓	✓	✓
N1	21.6	✓	✓*	✓*			
N2	12.4	✓	✓	✓			
N3	22.5	✓	✓	✓			
N4	21.5	✓	✓*	✓*			
N5	35.3	✓	✓	✓			
N6	16.6	✓	✓	✓			
O1	16.8		✓	✓			
O2	10.1		✓	✓			
O3	12.3		✓	✓			
O4	13.5		✓	✓			
O5	15.9		✓	✓			

Supplementary Table 1 | Experimental animals and groups. Notes: *, no EES; &, not recovered; ‡, failed to regain voluntary locomotion at 5 weeks; §, could not be performed due to health conditions.

Kinematic and kinetic gait parameters			
**Temporal features of gait**		**Joint angles and segmental oscillations**	
1	Cycle duration	32	Crest elevation angle amplitude
2	Cycle velocity	33	Hip elevation angle amplitude
3	Relative stance duration	34	Knee elevation angle amplitude
4	Swing duration	35	Ankle elevation angle amplitude
5	Relative phases alternation	36	Metatarsal elevation angle amplitude
6	Stance duration	37	Whole-limb elevation angle amplitude
**Limb endpoint trajectory**		38	Hip joint angle amplitude
7	Stride length	39	Knee joint angle amplitude
8	Step length	40	Ankle joint angle amplitude
9	3D endpoint path length	41	Metatarsal joint angle amplitude
10	Maximum backward position	42	Whole-limb abduction amplitude
11	Maximum forward position	43	Foot abduction amplitude
12	Relative step height	**Velocity**	
13	Maximum swing speed	44	Crest oscillation velocity amplitude
14	Relative timing of maximum speed during swing	45	Thigh oscillation velocity amplitude
15	Acceleration at swing onset	46	Leg oscillation velocity amplitude
16	Endpoint velocity	47	Foot oscillation velocity amplitude
17	Orientation of velocity vector at swing onset	48	Whole limb oscillation velocity amplitude
18	Time of foot dragging	**Limb coordination**	
19	Relative dragging duration	49	Temporal coupling between crest and thigh oscillations
20	Relative dragging duration terminal point	50	Temporal coupling between leg and thigh oscillations
21	Step height	51	Temporal coupling between leg and foot oscillations
**Stability**		52	Correlation between crest and thigh oscillations
22	Foot-pelvis relative position at stance onset	53	Correlation between leg and thigh oscillations
23	Stance width	54	Correlation between leg and foot oscillations
24	Maximum hip vertical position	55	Correlation between hip and knee oscillations
25	Minimal hip vertical position	56	Correlation between knee and ankle oscillations
26	Amplitude of hip vertical movement	57	Correlation between ankle and metatarsal oscillations
27	Variability of sagittal hip oscillations	58	Relative duration between crest and thigh angle minima
**Whole body movement**		59	Relative duration between thigh and leg angle minima
28	Pelvic center of mass forward motion	60	Relative duration between leg and foot angle minima
29	Pelvic center of mass mediolateral motion	61	Relative duration between crest and thigh angle maxima
30	Pelvic center of mass vertical motion	62	Relative duration between thigh and leg angle maxima
31	Pelvic center of mass 3D motion	63	Relative duration between leg and foot angle maxima

Supplementary Table 2 | **Kinematic and kinetic parameters for PC analysis.** Representative kinematic parameters used to assess gait quality in rats.

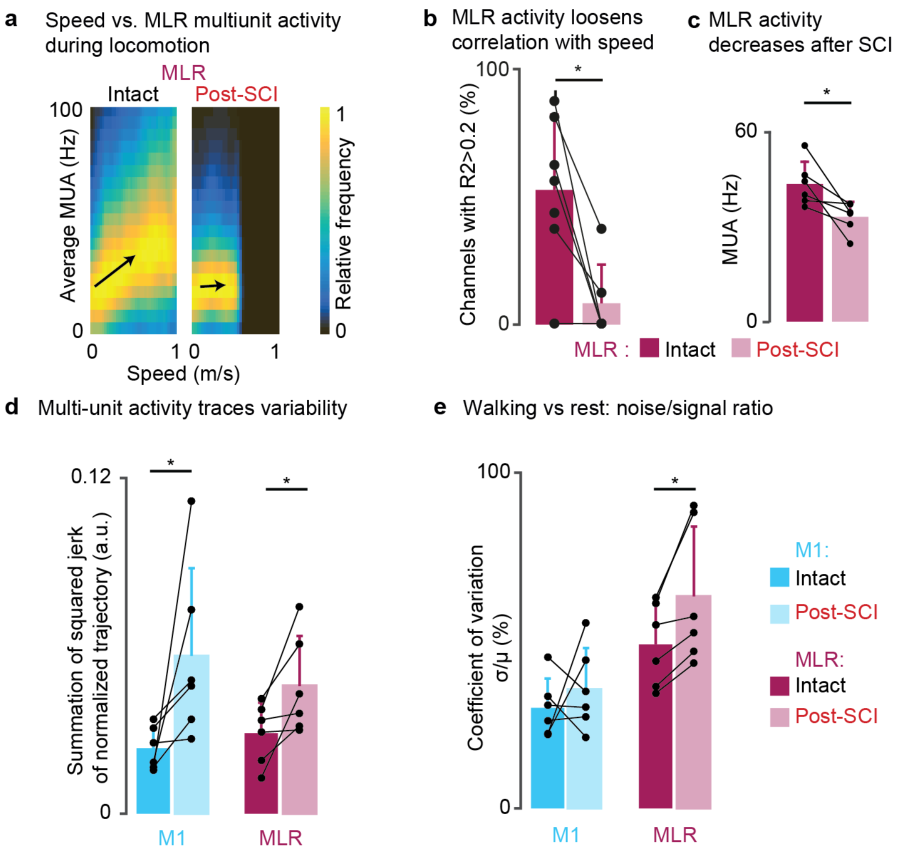

Supplementary Figure 1 | MLR and M1 Multi-unit temporal patterns analysis in intact and after SCI. **(a)** SCI rats display a smaller range of locomotor speeds, but no significant MLR modulation is found within these speeds. In intact rats higher speeds are associated with larger MLR firing rates. **(b)** Few MLR channels display multi-unit activity (MUA) correlation with speed after SCI (n=6 rats, p=0.031, paired, one-tailed Wilcoxon signed rank test). **(c)** MLR MUA decreases after SCI (n=6 rats, p=0.014, paired, one-tailed t-test). **(d)** M1 and MLR MUA variability increases after SCI (n=6 rats; p=0.036 M1; p=0.018 MLR; paired, one-tailed t-test). a.u., arbitrary units. **(e)** MLR noise-signal ratio in walking vs stance encoding increases after SCI (n=6 rats, p=0.012, paired, one-tailed t-test). Bar diagrams, mean±s.d.

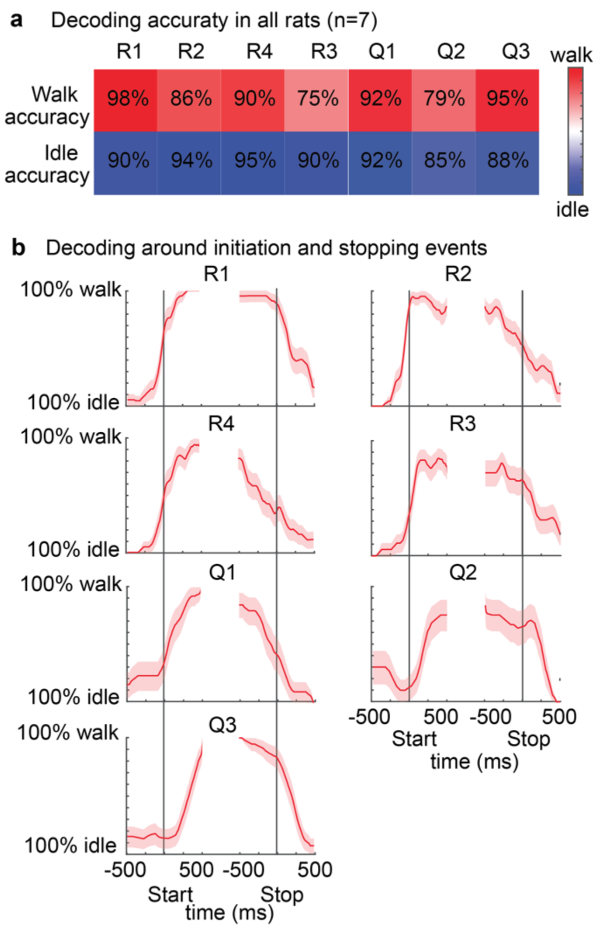

Supplementary Figure 2 | Online decoding accuracy in intact rats performing quadrupedal walking task (n=7). (a) Accuracy of the decoder in capturing walk and idle conditions, in stable states, >0.5s away from walk initiation and termination. **(b)** Decoding transition around walk initiation and termination. Walk initiation is defined as the first foot-off event and walk termination as the last foot strike event. Shaded areas, s.e.m.
