# Supplementary material for: Multi-pronged neuromodulation intervention engages the residual motor circuitry to facilitate walking in a rat model of spinal cord injury
Source: Nat Commun. 2021 Mar 26;12:1925. doi: 10.1038/s41467-021-22137-9 (PMC7997909; doi:10.1038/s41467-021-22137-9)
Supplement: Supplementary file 2 — Description of Additional Supplementary Files [file 41467_2021_22137_MOESM2_ESM.docx]

**Description of Additional Supplementary Files**

File Name: Supplementary Movie 1

Description: Representative examples of how various combinations of this multi-pronged neuromodulation therapy affect locomotion.
