# Supplementary material for: Multi-pronged neuromodulation intervention engages the residual motor circuitry to facilitate walking in a rat model of spinal cord injury
Source: Nat Commun. 2021 Mar 26;12:1925. doi: 10.1038/s41467-021-22137-9 (PMC7997909; doi:10.1038/s41467-021-22137-9)
Supplement: Supplementary file 4 — Reporting Summary [file 41467_2021_22137_MOESM4_ESM.pdf]

nature research

Corresponding author(s): Courtine Gregoire

Last updated by author(s): Sep 18, 2019

Reporting Summary

Nature Research wishes to improve the reproducibility of the work that we publish. This form provides structure for consistency and transparency in reporting. For further information on Nature Research policies, see Authors & Referees and the Editorial Policy Checklist .

Statistics

For all statistical analyses, confirm that the following items are present in the figure legend, table legend, main text, or Methods section.

n/a	Confirmed	
☐	☒	The exact sample size (*n*) for each experimental group/condition, given as a discrete number and unit of measurement
☐	☒	A statement on whether measurements were taken from distinct samples or whether the same sample was measured repeatedly
☐	☒	The statistical test(s) used AND whether they are one- or two-sided<br>*Only common tests should be described solely by name; describe more complex techniques in the Methods section.*
☐	☒	A description of all covariates tested
☐	☒	A description of any assumptions or corrections, such as tests of normality and adjustment for multiple comparisons
☐	☒	A full description of the statistical parameters including central tendency (e.g. means) or other basic estimates (e.g. regression coefficient) AND variation (e.g. standard deviation) or associated estimates of uncertainty (e.g. confidence intervals)
☐	☒	For null hypothesis testing, the test statistic (e.g. *F*, *t*, *r*) with confidence intervals, effect sizes, degrees of freedom and *P* value noted<br>*Give P values as exact values whenever suitable.*
☒	☐	For Bayesian analysis, information on the choice of priors and Markov chain Monte Carlo settings
☒	☐	For hierarchical and complex designs, identification of the appropriate level for tests and full reporting of outcomes
☒	☐	Estimates of effect sizes (e.g. Cohen's *d*, Pearson's *r*), indicating how they were calculated

Our web collection on statistics for biologists contains articles on many of the points above.

Software and code

Policy information about availability of computer code

Data collection	Online data collection was performed using the OpenEx platform by Tucker-Davis Technology and Matlab (v2018b) custom code for decoder calibration. To review our code, we provide sample M1 data recorded during a sample overground locomotion trial and the Matlab code used to calibrate the decoder (see section Cortex-midbrain interface in Methods). Imaris 9.6 (Oxford instruments) was used for c-fos density analysis. Mixed model analyses of this data was carried out using the lme4 package in R, and the corrected R2 calculated using the MuMIn package
Data analysis	Offline data analysis was perfomed in Matlab 2018b. Sample raw data of M1 and MLR activity are made available, as well as the routine used to extract M1 and MLR activation. https://github.com/multipronged-neuromodulation/M1-MLR

For manuscripts utilizing custom algorithms or software that are central to the research but not yet described in published literature, software must be made available to editors/reviewers. We strongly encourage code deposition in a community repository (e.g. GitHub). See the Nature Research guidelines for submitting code & software for further information.

Data

Policy information about availability of data

All manuscripts must include a data availability statement. This statement should provide the following information, where applicable:

- Accession codes, unique identifiers, or web links for publicly available datasets
- A list of figures that have associated raw data
- A description of any restrictions on data availability

Complete source data are provided with this paper. Data that support the findings will be made available upon reasonable request to the corresponding author.

Field-specific reporting

Please select the one below that is the best fit for your research. If you are not sure, read the appropriate sections before making your selection.

- [x] Life sciences
- [ ] Behavioural & social sciences
- [ ] Ecological, evolutionary & environmental sciences

For a reference copy of the document with all sections, see nature.com/documents/nr-reporting-summary-flat.pdf

Life sciences study design

All studies must disclose on these points even when the disclosure is negative.

Sample size	Sample sizes were initially chosen based on previous physiological studies using similar animal models to guarantee statistical relevance based on a similar effect size (see van den Brand et al., 2012; Asboth et al., 2018). Rehabilitation procedures require time-consuming procedures and, as a consequence, only a maximum of n=6 animals can be trained within the same timeframe. Due to reviewer comments a new cohort of n=6 animals was added to the original dataset in order to investigate comparisons of individual stimulation types.
Data exclusions	All experiment performed are reported in Table T1. After injury, one subject was excluded, untrained and untested, due to health conditions. Animals that did not recover were not excluded but rather treated separately and equally visualized in figure 7.
Replication	All tested conditions were repeated across multiple trials and the results averaged to obtain a single-subject mean performance (approximately 25 gait cycles per condition). All replications of the tested effects are presented within the article and are described in Table T1.<br>In the Methods section "Evaluation and characterization of midbrain stimulation" we report the implant success rates, which was 100% for commercial electrodes.
Randomization	Animals are not divided into groups. Testing conditions were presented at random within a session.
Blinding	Grimace stress scoring was performed blinded by 2 independent non-author investigators. For kinematic analysis of all locomotion data under all treatment conditions blinding was not possible (and irrelevant, due to behavioral changes across conditions), however video tracking and processing of neural data are highly automatized tasks.

Reporting for specific materials, systems and methods

We require information from authors about some types of materials, experimental systems and methods used in many studies. Here, indicate whether each material, system or method listed is relevant to your study. If you are not sure if a list item applies to your research, read the appropriate section before selecting a response.

Materials & experimental systems

n/a	Involved in the study	
[ ]	[x]	Antibodies
[x]	[ ]	Eukaryotic cell lines
[x]	[ ]	Palaeontology
[ ]	[x]	Animals and other organisms
[x]	[ ]	Human research participants
[x]	[ ]	Clinical data

Methods

n/a	Involved in the study	
[x]	[ ]	ChIP-seq
[x]	[ ]	Flow cytometry
[x]	[ ]	MRI-based neuroimaging

Antibodies

Antibodies used	All information about antibodies is described in Method section. Briefly, we used anti-GFAP (1:1000, Dako, USA; catalogue number GA524), anti-choline acetyltransferase (1:100; Millipore, USA; catalogue number AB144P), anti-c-Fos (1:100; Synaptic Systems, Germany; catalogue number 226 003) as primary antibodies. secondary antibodies used were as follows: donkey anti-goat Alexa fluor 647, (Life Technology A2 1432), goat anti-rabbit Alexa fluor 488 (Life technology A-11008).
Validation	We used commercial antibodies. All of them were quality controlled by the manufacturer. Additionally, each of these antibodies has been previously validated and used extensively in our own laboratory in numerous rat studies.

Animals and other organisms

Policy information about studies involving animals; ARRIVE guidelines recommended for reporting animal research

Laboratory animals	Adult (10-14 weeks at beginning of study) Lewis rats, Females.
Wild animals	No wild animals were used in this study

Field-collected samples	No field samples were collected for this study
Ethics oversight	All experimental procedures were approved by the Veterinary Office of the Cantons of Vaud and Geneva, Switzerland.

Note that full information on the approval of the study protocol must also be provided in the manuscript.
